# Computational design of foldable origami-based compressive ultrasound sensing

**DOI:** 10.1038/s41598-026-37215-5

**Published:** 2026-01-31

**Authors:** Nicolas Hochuli, Tim Wünsch, Weiye Li, Xiaohan Han, Daniel Razansky, Tino Stanković, Héctor Estrada

**Affiliations:** 1 Engineering Design and Computing Laboratory, Department of Mechanical and Process Engineering, ETH Zurich, Zurich, Switzerland; 2 Institute of Pharmacology and Toxicology and Institute for Biomedical Engineering, Faculty of Medicine, University of Zurich, Zurich, Switzerland; 3 Department of Information Technology and Electrical Engineering, Institute for Biomedical Engineering, ETH Zurich, Zurich, Switzerland

**Keywords:** Engineering, Materials science, Mathematics and computing, Physics

## Abstract

Ultrasound imaging is an essential part of the modern clinical routine. However, its dependence on costly multichannel electronics limits its use in chronic monitoring of disease. Single-detector compressed-sensing approaches have been proposed to simplify the signal acquisition pipeline, but they suffer from reduced acoustic sensitivity due to reliance on multiple scattering topologies. We propose foldable origami structures with built-in ultrasound sensing capabilities for single-pixel imaging that increase the acoustic sensitivity by leveraging a foldable transducer geometry. By detecting ultrasound fields at various origami folding states, target images in two- and three-dimensions are recovered using model-based reconstruction techniques. We simulated the Foldable Origami-based Compressive Ultrasound Sensing (FOCUS) concept and inverse designed the origami geometry for maximum imaging performance. We quantified the performance of the FOCUS concept with the reconstruction accuracy of synthetic target images including point-scatterers and vessel-like structures, reaching an average structure similarity index measure of 0.63 and $\:{L}_{2}$ error of 11.89. We showed that the optimized FOCUS pattern remains effective even when exposed to geometric distortions and electrical noise. Our approach can tailor the FOCUS design to various targets, scales, and applications, potentially transforming ultrasound imaging devices through miniaturized single-channel electronics.

**Supplementary Information:**

The online version contains supplementary material available at 10.1038/s41598-026-37215-5.

## Introduction

Ultrasound-mediated imaging is widely used in clinics to observe biological processes within our bodies. Traditional clinical systems employ hundreds of piezoelectric transducers to generate real-time, two-dimensional images, relying on bulky and expensive multichannel electronics. Recent advancements in ultrasound technology, such as three-dimensional ultrasound^1,2^ and ultrasound localization microscopy^3–6^, have only increased the data throughput and number of channels needed from electronic acquisition systems. Consequently, efforts are being made to reduce the complexity of electronic hardware by compensating for any loss in imaging performance through software complexity, utilizing deep learning and compressed sensing algorithms^7–11^.

Pushed to their extreme, compressed sensing methods enable single-transducer imaging in pulse-echo ultrasound and optoacoustic scenarios. This is achieved by the modulation of the ultrasound field to create a distinct fingerprint for each point in space. Existing examples utilize either scattering masks^12–15^ or multiple scattering cavities^16–19^. However, the proposed designs are not compact and its reliance on ultrasound wave scattering hampers its sensitivity.

Origami offers a novel approach to designing ultrasound transducers. Transducers realized as origami-based structures could be designed to precisely direct the acoustic energy based on the spatial configurations of their panels, while being controlled by a comparatively low number of degrees of freedom. Driven by a similar rationale, origami transducers with distinct focusing patterns have been proposed and characterized in several recent publications^20–22^. However, origami’s most remarkable ability to transform from a flat sheet into various predetermined shapes^23–25^ has barely been explored for multichannel ultrasound focusing devices^26,27^, and remains unexplored for single-channel imaging systems. Although the literature reports on the successful use of Miura-ori and the rotationally symmetric “flasher” origami^28^ for transducer design, the underlying geometries of crease patterns and their established kinematic behavior^28,29^ are used as is, without attempting to computationally explore the design space of origami-based transducers to enhance their performance. This leaves the full potential of their spatial configurations unexplored, limiting the design space of feasible origami-based transducers throughout the design process.

Motivated by the potential of origami for transducer design, here we propose a piezoelectric Foldable Origami-based Compressive Ultrasound Sensing (FOCUS) concept that modulates the ultrasound field through its shape-changing motion to achieve single-channel imaging. We computationally explore the design space of a Single Degree of Freedom (SDOF) rigidly flat foldable quadrilateral origami meshes^30^ and their spatial configurations by developing a simulation-driven inverse design framework. To develop the framework first we propose an ultrasound image reconstruction algorithm given multiple folding states of an origami transducer using a total variation-regularized least squares reconstruction. Second, we extend the mutual coherence measure^31^, originally developed to serve as a lower bound on the sparsity of imaging targets in compressed sensing, to introduce the Minimum Coherence Principle (MCP) as a metric to evaluate the ultrasound imaging performance across multiple folding states of a FOCUS transducer. Third, we show that the MCP serves as a computationally efficient objective function for the inverse design of the FOCUS transducer and demonstrate the superior generalizability of MCP for two-and three-dimensional ultrasound imaging in comparison to a naïve objective function based on a statistical evaluation of the transducer’s reconstruction performance on a synthetic training set. Finally, the results demonstrate the potential of the FOCUS concept for ultrasound imaging on a diverse test set including point-scatterers and vessel-like target images, while also highlighting its robustness against noise and geometric imperfections. The inverse-design framework proposed in this work offers a generic approach to support the computational design of origami-based FOCUS transducers and can be developed further for different imaging scenarios, accommodating different targets, depths, and resolutions.

## Results

### Ultrasound image reconstruction using multiple folding states

**Fig. 1:**
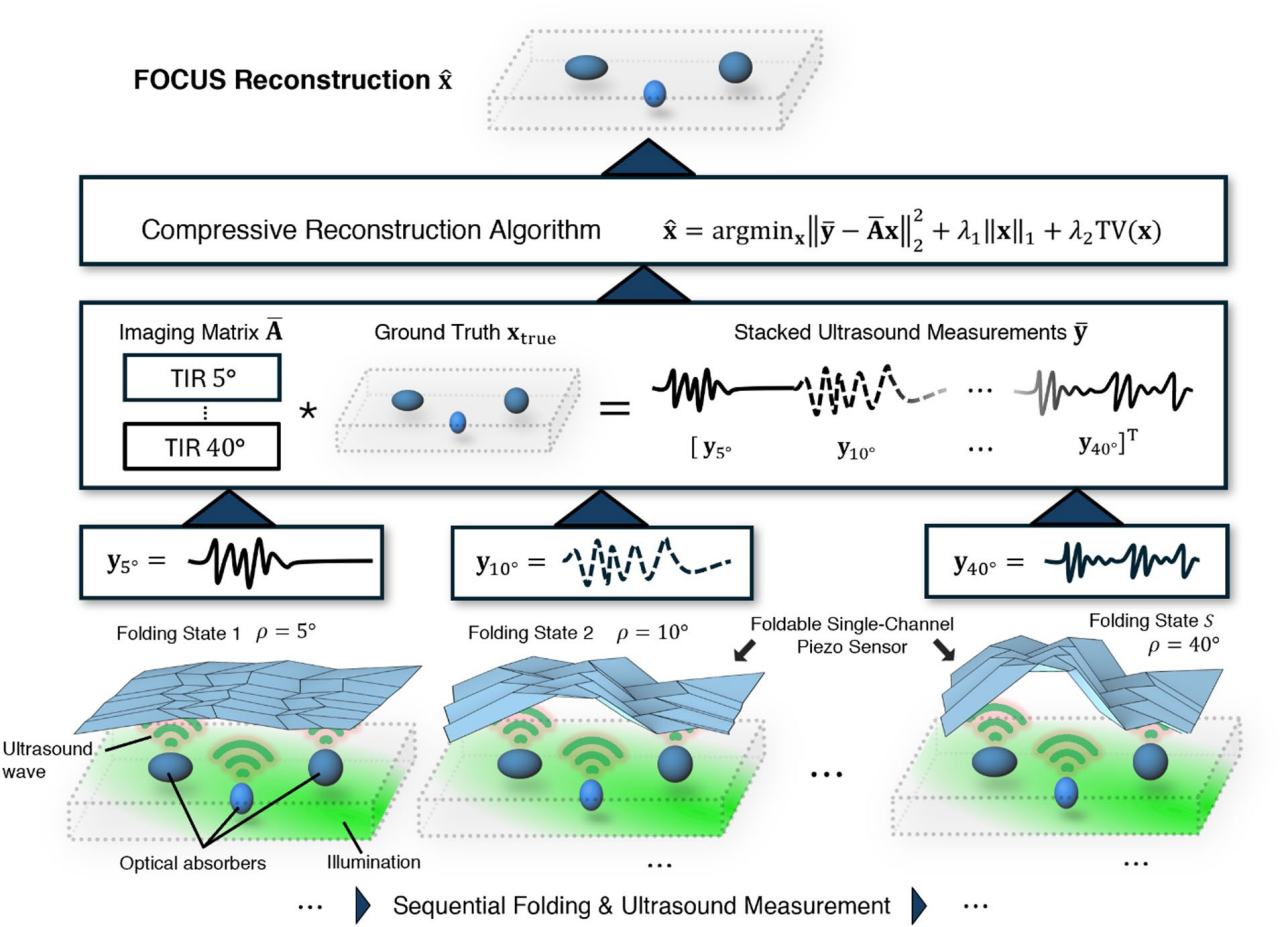
Foldable origami-based compressive ultrasound sensing (FOCUS). Schematic of the proposed FOCUS concept (bottom to top). The SDOF origami is actuated to different folding states governed by their driving angles ($\:\rho\:$), modulating the detected optoacoustic ultrasound field and encoding spatial information via the total impulse response (TIR). By acquiring incoherent measurements across multiple folding states $\:{\mathbf{y}}_{\rho\:}$, a compressive reconstruction algorithm (see Methods for details) demodulates and reconstructs the underlying tissue structure $\:\widehat{\mathbf{x}}$ using single-channel electronics.

To reconstruct an ultrasound image $\:\mathbf{x}\in\:\:{\mathbb{R}}^{{N}^{2}}$ in a square field-of-view (FOV) with $\:N\times\:N$ pixels using multiple folding states of an origami transducer, we solve a linear system of equations $\:\overline{\mathbf{y}}\mathrm{=}\overline{\mathbf{A}}\mathbf{x}$ with, in general, infinitely many solutions. Here, $$\:\overline{\mathbf{y}}={\left[\begin{array}{ccc}{\mathbf{y}}_{1}&\:\dots\:&\:{\mathbf{y}}_{S}\end{array}\right]}^{\mathrm{T}}\in\:{\mathbb{R}}^{ST}$$ represents the stacked ultrasound measurements in terms of time-series data where each $\:{\mathbf{y}}_{i}$ holds $\:T\:$zero-padded samples. $$\:\overline{\mathbf{A}}={\left[\begin{array}{ccc}{\mathbf{A}}_{1}&\:\dots\:&\:{\mathbf{A}}_{S}\end{array}\right]}^{\mathrm{T}}\in\:{\mathbb{R}}^{ST\times\:{N}^{2}}$$ represents the stacked imaging matrices at $\:S$ distinct folding states (Fig. 1) that hold the total impulse response (TIR) of the transducer, defined by the geometry of the origami surface at each folding state and the electrical impulse response of the thin piezoelectric material vibrating in a thickness mode configuration. The TIRs are obtained through computational ultrasound simulation equivalent to a hydrophone calibration in a physical setup. The image $\:\widehat{\mathbf{x}}$, proportional to the target’s light absorption in optoacoustic imaging, can be estimated using $\:{L}_{2}$-error minimization combined with$\:{\text{}L}_{1}$- and total-variation (TV) regularization (see Methods for details).

To construct a useful imaging matrix, the responses from different voxels in the field-of-view (FOV) to a distinct folding state should ideally be linearly independent. This translates to achieving minimal coherence in the basis of the matrix $\:\overline{\mathbf{A}}$, which we define as the Minimum Coherence Principle (MCP).

### Design representation

**Fig. 2:**
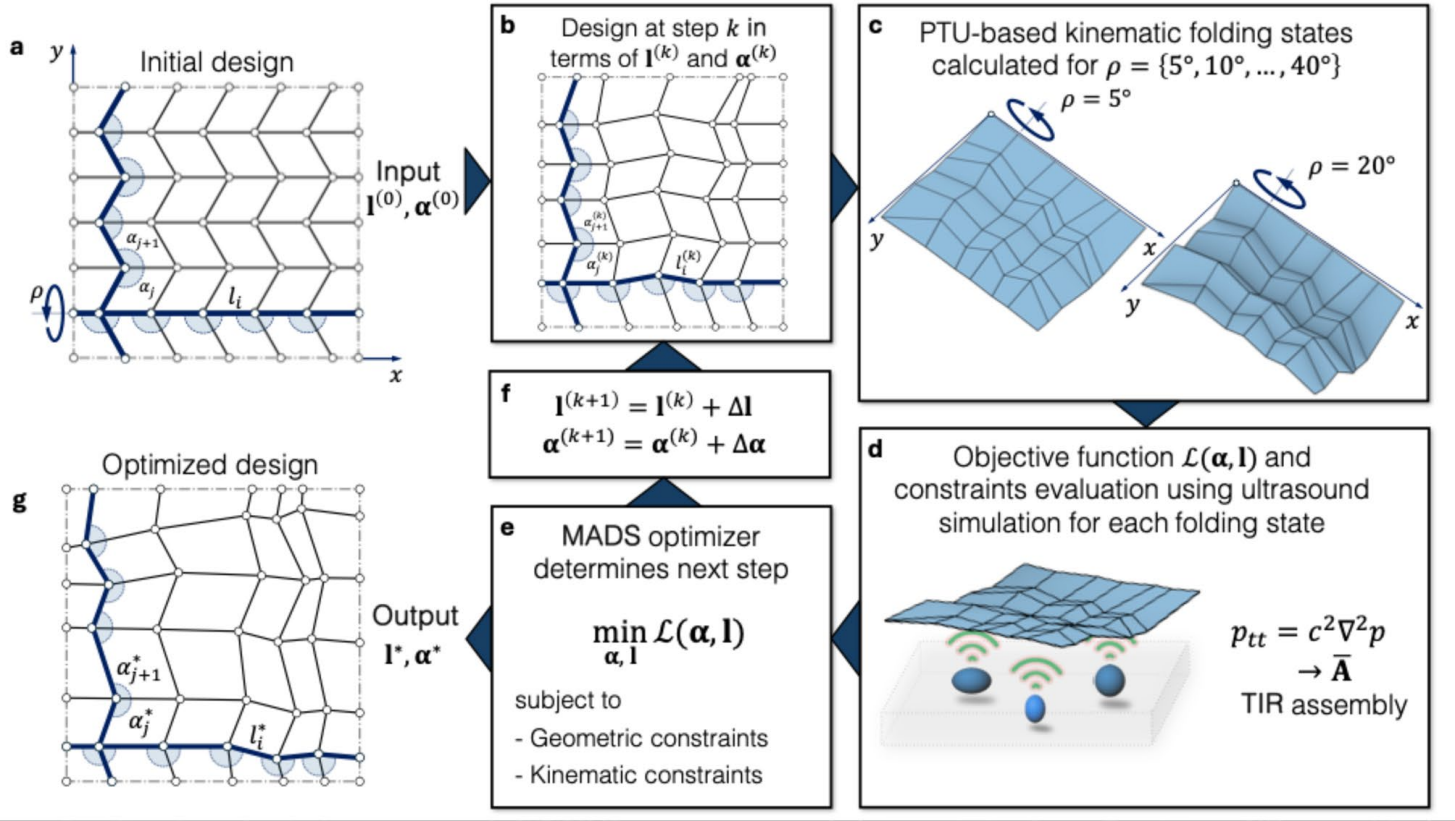
Optimization procedure for the FOCUS crease pattern design. (**a**) The procedure is initialized using the known Miura-ori pattern. The pattern is represented in terms of the governing sector angles $\:{\boldsymbol{\alpha\:}}^{\left(0\right)}$ and crease lengths $\:{\boldsymbol{l}}^{\left(0\right)}$ within the L-shaped motif, shown as blue sectors and thick edges, respectively. (**b**) Based on this parametric representation, the complete RFFQM is generated according to the panel compatibility procedure. (**c**) The PTU-based kinematic simulation is used for the evaluation of the origami in eight distinct folded states given by the driving angles $\:\rho\:\in\:\left\{5^\circ\:,10^\circ\:,\dots\:,40^\circ\:\right\}.$ The folded configuration is aligned with a global coordinate system (see Methods for details). (**d**) The objective function evaluation performs a simulation of ultrasound propagation and retrieves the TIR of the transducer that characterizes the imaging performance. (**e**,** f**) The MADS algorithm determines the next iteration in the optimization, subject to a range of constraints on the origami geometry and kinematics. (**g**) When the stopping criteria for the optimization are met (see Methods for details), the optimized crease pattern is returned in terms of its design variables $\:{\boldsymbol{l}}^{*},{\boldsymbol{\alpha\:}}^{*}$.

To computationally design a FOCUS transducer, we model the underlying design using the paradigm of rigidly and flat-foldable quadrilateral mesh origami^30^ (RFFQM). RFFQM are a generalization of the well-known Miura-ori pattern and a suitable choice for the FOCUS design due to three reasons: First, the complete origami crease pattern can be represented in terms of a so-called L-shaped motif (Fig. 2a). Here, the motif is parametrized using a 30-dimensional vector of sector angles $\:\boldsymbol{\upalpha\:}$ measured between crease lines in the motif and the lengths $\:\mathbf{l}$ of the crease lines in the motif (see Fig. 2a and Methods for details). The choice of a 30-dimensional design space for the RFFQM is motivated by the fact that most black-box optimizers suffer from poor convergence for larger dimensionalities. Second, the generally small out-of-plane deformation across the folding states preserves a practical formfactor during mechanical actuation. Third, RFFQM allow for SDOF rigid folding motion of the origami controlled by the scalar driving angle $\:\rho\:$, while still enabling a complex kinematic motion to facilitate the MCP. Here, the rigid flat-foldability aspect is crucial for a stress-free folding motion of the origami without introducing bending in the faces.

### Inverse design

To obtain the folding states of a candidate RFFQM depending on $\:{\boldsymbol{\upalpha\:}}^{\left(k\right)},{\mathbf{l}}^{\left(k\right)}$at step $\:k$ in the inverse design loop (Fig. 2B), we employ the Principle of Three Units^32^ (PTU) which models SDOF rigidly foldable crease patterns as directed graphs and applies the spherical law of cosines at each internal vertex to analytically calculate the folding kinematics. Here, the PTU is applied to calculate spatial configurations of the candidate RFFQM using a sequence of eight driving angles $\:\rho\:$ in the set $\:\left\{5^\circ\:,10^\circ\:,\dots\:,40^\circ\:\right\}$ (Fig. 2c). The number of folding states influences the subsequent imaging performance of FOCUS, where more folding states tendentially improve the performance at the cost of a higher computational burden (see Methods and Supp. Fig. S1.). The choice of eight folding states is deemed to present a good trade-off. To ensure a valid mountain-valley assignment of the pattern, i.e. whether the crease lines fold concavely or convexly with respect to the flat state, the standard Miura-ori based assignment^33^ is employed. Recognizing that origami are invariant under isometric transformations, we align a global frame of reference with the origami geometry (Fig. 2c, see Methods for details) and scale it such that its initial folded state at $\:\rho\:\mathrm{=}5^\circ\:$ does not exceed a projected area of $\:400\mathrm{m}\mathrm{m}^{2}$ in the $\:xy$-plane, allowing the transducer to remain within a practical formfactor. The size influences the transducer’s central frequency.

The ultrasonic pressure field for each folding state of the origami (Fig. 2d, see Methods for details) is simulated with a realistic excitation pulse to assemble the TIR $\:{\mathbf{A}}_{i}$of the transducer in the $\:i$-th folding state.

Given $\:\overline{\mathbf{A}}$, the objective function to optimize the FOCUS transducer is stated under the assumption that the imaging performance directly relates to the MCP, i.e. how mutually incoherent the basis of $\:\overline{\mathbf{A}}$ is (Fig. 2e). The objective function is quantified as a scalar function $\:\mathcal{L}\left(\boldsymbol{\upalpha\:},\mathbf{l}\right)$ that depends only on the design variables $\:\boldsymbol{\upalpha\:},\mathbf{l}$ of the underlying L-shaped motif of the pattern that directly influences $\:\overline{\mathbf{A}}(\boldsymbol{\upalpha\:},\mathbf{l})$. Thus, we formulate the inverse design as an optimization problem, in which the MCP-based objective function in Eq. (1), stated as the squared Frobenius norm of the upper triangular part of the column-wise inner products of $\:\stackrel{\sim}{\mathbf{A}}$, i.e. the symmetric Gram matrix $\:{\stackrel{\sim}{\mathbf{A}}}^{\mathrm{T}}\stackrel{\sim}{\mathbf{A}}$, with exclusion of the diagonals, is minimized:

1 $$\:\mathcal{L}\left(\boldsymbol{\upalpha\:},\mathbf{l}\right)=100\frac{2}{{{N}^{2}(N}^{2}-1)}{||{\mathrm{t}\mathrm{r}\mathrm{i}\mathrm{u}(\stackrel{\sim}{\mathbf{A}}}^{\mathrm{T}}\stackrel{\sim}{\mathbf{A}})-{\mathrm{d}\mathrm{i}\mathrm{a}\mathrm{g}(\stackrel{\sim}{\mathbf{A}}}^{\mathrm{T}}\stackrel{\sim}{\mathbf{A}})||}_{F}^{2}$$

Here $\:\stackrel{\sim}{\mathbf{A}}\:$is the column-wise $\:{L}_{2}$ normalized TIR $\:\stackrel{-}{\mathbf{A}}\mathrm{,}\text{}{N}^{2}$ is the number of pixels in the FOV, $\:\mathrm{t}\mathrm{r}\mathrm{i}\mathrm{u}$ represents the upper triangular part and $\:\mathrm{d}\mathrm{i}\mathrm{a}\mathrm{g}$ the diagonal part of the matrices, respectively. The scaling factor $\:\frac{2}{{{N}^{2}(N}^{2}\mathrm{-}1)}$ is proportional to the number of elements in the upper triangular Gram matrix with exclusion of the diagonals and multiplied by a factor 100 for better readability. The reasoning for using the Gram matrix to quantify the linear independence of the TIR comes from the inner product space that is established through $\:{\stackrel{\sim}{\mathbf{A}}}^{\mathrm{T}}\stackrel{\sim}{\mathbf{A}}$. Here, off-diagonal values near zero indicate incoherent bases. Only the diagonal elements, representing self-inner products, should remain nonzero.

Alongside objective function in Eq. (1) a set of four geometric constraints is defined that ensures ease of actuation and restricts the folding motion of the transducer design (Fig. 2e) to a predefined space (see Methods for details).

**Fig. 3:**
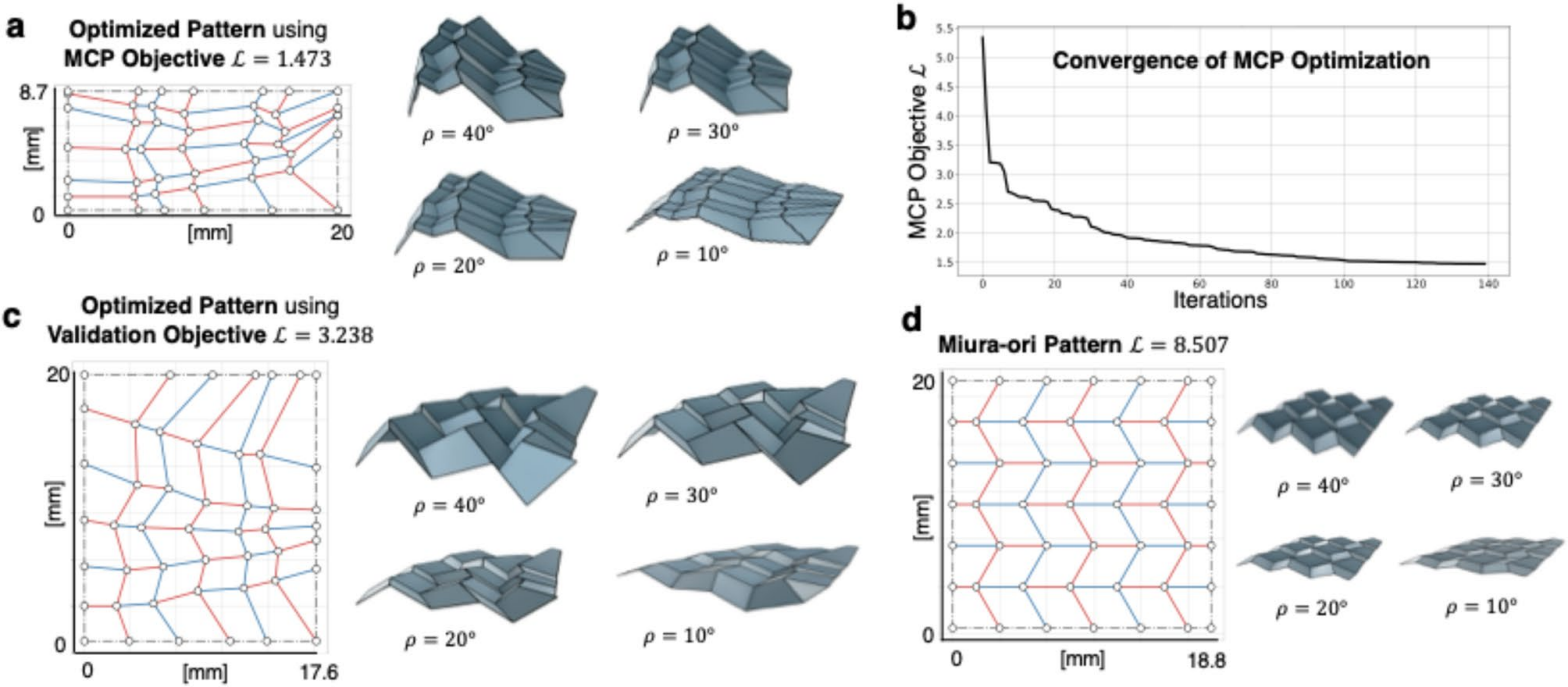
Optimized crease patterns and corresponding folded configurations for the FOCUS transducer. Three different FOCUS crease patterns and folded states for (**a**) MCP-optimized (Eq. (1)), (**c**) validation-optimized (Eq. (2)), and (**d**) Miura-ori (see Fig. S3 for more detailed visualizations). The colors of the crease lines represent mountain (in red) and valley (in blue) assignments. (**b**) Convergence plot for the optimization in (**a**), displaying only the iterations that reduce the objective function value; non-decreasing steps are omitted.

Assuming a non-convex design space under the proposed objective function formulation, we apply the Mesh Adaptive Directed Search (MADS) algorithm which is a derivative free black box function optimizer based on mesh-adaptive directed search^34^ to minimize Eq. (1) and iteratively design the optimal crease pattern (Fig. 2f, see Methods for details). The selection of the MADS algorithm is justified by providing advantages over other meta-heuristics in optimizing continuous design spaces with up to 30 dimensions and an expensive objective function. These advantages include the utilization of surrogate models for intermediate optimization iterations, the application of Latin-hypercube sampling to explore the design space effectively and less sensitivity to tunable parameters. This process generates the MCP-optimized crease pattern design (Fig. 3a, Supp. Movie. S1 and S2, Supp. Data S1 and Fig. S3a), showing convergence after hundreds of iterations (Fig. 3b).

### Design validation

To empirically ensure that the MCP-based optimization in Eq. (1) is relying on reasonable assumptions, we validate the FOCUS design by using a direct and more naïve objective $\:{\mathcal{L}}_{\mathrm{v}\mathrm{a}\mathrm{l}}\left(\boldsymbol{\upalpha\:},\mathbf{l}\right)$. Thus, an optimized transducer design using the validation objective function $\:{\mathcal{L}}_{\mathrm{v}\mathrm{a}\mathrm{l}}\left(\boldsymbol{\upalpha\:},\mathbf{l}\right)$ in Eq. (2) serves as a benchmark for the imaging performance of the MCP-based result. $\:{\mathcal{L}}_{\mathrm{v}\mathrm{a}\mathrm{l}}\left(\boldsymbol{\upalpha\:},\mathbf{l}\right)$ is based on a statistical evaluation of the transducer’s ability to reconstruct a synthetic training set of $\:K\mathrm{=}28$ imaging targets $\:{\mathbf{x}}_{\mathrm{t}\mathrm{r}\mathrm{u}\mathrm{e},i}$ in the tissue (see Fig. S2 for the dataset):

2 $$\:{\mathcal{L}}_{\mathrm{v}\mathrm{a}\mathrm{l}}\left(\boldsymbol{\upalpha\:},\mathbf{l}\right)=\frac{1}{K}\sum\:_{i=1}^{K}{||{\mathbf{x}}_{\mathrm{t}\mathrm{r}\mathrm{u}\mathrm{e},i}-{\widehat{\mathbf{x}}}_{i}||}_{2}^{2}$$

where, $\:{\widehat{\mathbf{x}}}_{i}$ is the estimated pixel reconstruction obtained by solving $\:{\stackrel{-}{\mathbf{y}}}_{i}=\stackrel{-}{\mathbf{A}}{\mathbf{x}}_{i}$ using the total variation least squares approach (see Methods for details). Choosing the number of samples $\:K$ in the training set presents a trade-off between the generalizability and evaluation times of the objective. A small training set increases the risk of overfitting, while a large $\:K$ leads to long evaluation times and, consequently, fewer possible optimization iterations, hindering the convergence within a given timeframe. The resulting optimized crease pattern using the validation objective is shown in Fig. 3c with detailed information available in the Supplementary Information (Data S2 and Fig. S3b). Figure 3c reports a higher minimum coherence value $\:\mathcal{L}\left(\boldsymbol{\upalpha\:},\mathbf{l}\right)$ for the optimized pattern using Eq. (2) compared to the results in Fig. 3a using the MCP-based objective function in Eq. (1).

### FOCUS performance

**Fig. 4:**
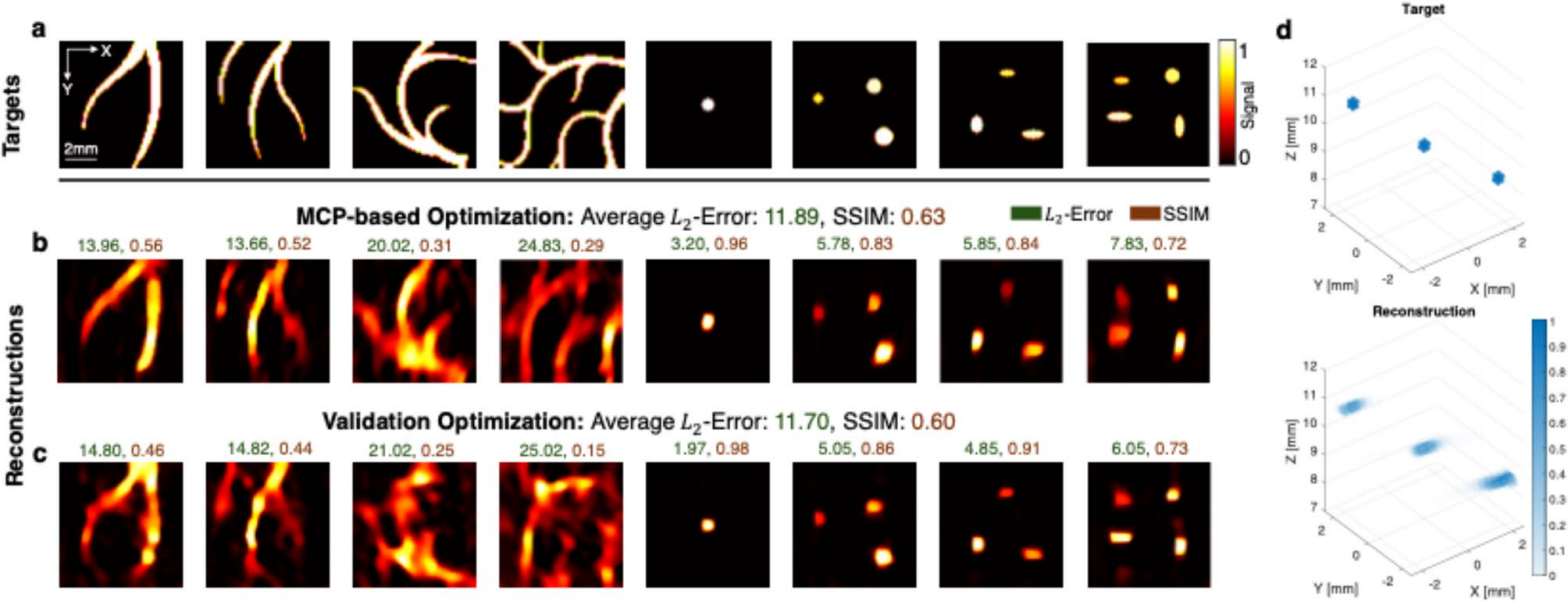
Evaluation of FOCUS imaging capabilities across different 2D and 3D target configurations. (**a**) Visualization of eight 2D imaging targets in the test set and reconstructions of the targets using (**b**) the MCP-optimized and (**c**) validation-optimized pattern. $\:{L}_{2}$-errors and SSIM values are displayed above each reconstruction by color labels. The average reconstruction performance is displayed in the panel title. (**d**) Three-dimensional reconstruction with the MCP-optimized pattern. The reconstruction yields an $\:{L}_{2}$-error of 10.86 and an SSIM of 0.903.

We first tested the FOCUS reconstruction accuracy on a test set of eight different imaging targets (Fig. 4a) and compared the outputs of the MCP-optimized (Fig. 4b) and validation-optimized (Fig. 4c) patterns. The test set consists of a mixture of point-scatterers and vessel-like fingerprints while the latter was not present in the training set (Supp. Fig. S2) of the validation objective. The exclusion of vessel-like images from the training set of the validation objective is justified to analyze the generalizability of both approaches to previously unseen imaging targets. Although the average $\:{L}_{2}$-error is lower for the validation-optimized pattern (11.70 < 11.89), this stems from the very high accuracy on the point-scatterers as these targets were similarly present in the training dataset, implying limited generalizability of the validation-optimized pattern. In contrast, the accuracy of the reconstruction on the vessel-like structure shows more robustness using the proposed MCP optimized pattern, indicating better generalizability and imaging performance compared to the validation objective. This is also reflected in the average structural similarity index measure^35^ (SSIM, higher is better), which is higher for the MCP-optimized pattern. Unlike during the inverse design stage, a set of 20 folding states is used for the reconstruction of the test set (see Fig. S1 for a sensitivity analysis on different combinations and number of folding states for reconstruction). Although the optimization process does not explicitly model 3D imaging capabilities, the reconstruction of a simple 3D target (Fig. 4d) demonstrates an effective reconstruction performance using the MCP-optimized pattern.

Furthermore, after repeating the optimization using $\:\mathcal{L}$ and $\:{\mathcal{L}}_{\mathrm{v}\mathrm{a}\mathrm{l}}$ for six distinct random seeds in the MADS algorithm (see Methods), the average performance on the test set (Table 1) of the resulting MCP-optimized patterns outperformed the validation-optimized pattern in both the $\:{L}_{2}$-Error and SSIM. This statistical analysis with consistently outperforming average metrics for the MCP-based objective, further strengthens the claimed superior robustness and better imaging performance of the MCP-based approach, compared to the validation-based approach.

**Table 1:** Averaged repeated optimization metrics. Summary of the average metrics ($\:{L}_{2}$-error and SSIM) for the MCP- and validation-based optimization results on the test set across six different initial seeds of the MADS algorithm. (see Supp. Table S1).

Metrics	MCP-based		Validation-based	
	$\:{L}_{2}$-error	SSIM	$\:{L}_{2}$-error	SSIM
Average	**12.51**	**0.602**	12.647	0.572
Standard deviation	0.37	0.025	0.723	0.021

**Fig. 5:**
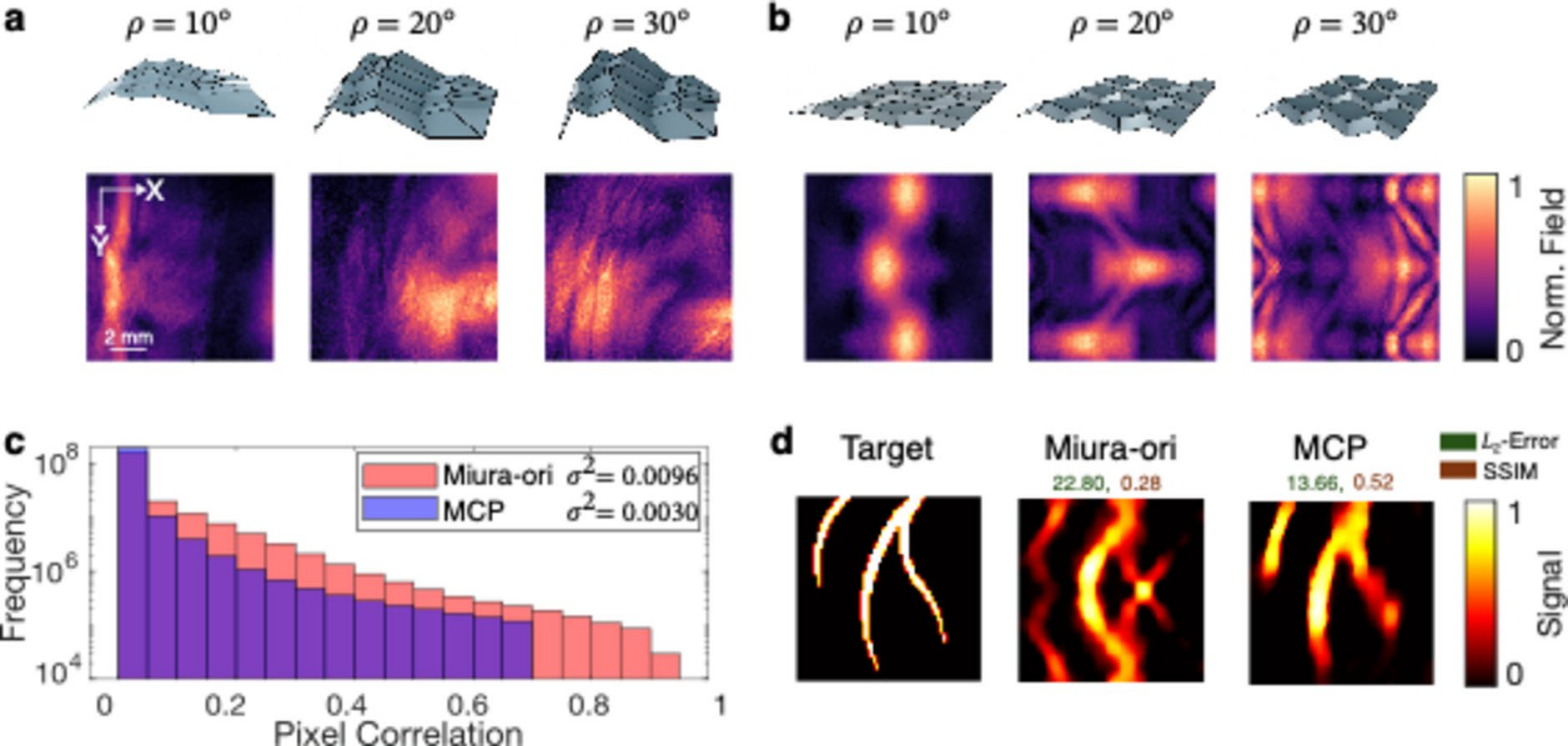
FOCUS imaging performance of MCP-optimized transducer versus Miura-ori transducer. Folding states and acoustic sensitivity field of (**a**) MCP-optimized and (**b**) Miura-ori. (**c**) Correlation distribution in the TIR. (**d**) FOCUS image reconstruction of a vessel-like target. $\:{L}_{2}$-error and SSIM indicated by color labels.

We also evaluate the characteristics of the FOCUS acoustic sensitivity field, defined as the temporal evolution of the TIR within the FOV which reflects the transducer’s responsiveness to specific points in space, for the MCP-optimized (Fig. 5a) pattern and compare it to a standard Miura-ori (Fig. 5b) pattern (see Supp. Information 5 and Supp. Movie S1 for details on the acoustic sensitivity field). Ideally, the acoustic sensitivity field evolution is maximally aperiodic to achieve the best imaging performance. The sensitivity field of the Miura-ori pattern exhibits a high periodicity as reflected in the pattern itself, whereas the MCP-optimized pattern shows a more irregular field by design. We quantify the irregularity with the sensitivity field correlation (Fig. 5c), defined as the histogram of the absolute values of the correlation matrix of the TIR^12^, which is strongly connected to the MCP’s objective function. The MCP-optimized FOCUS shows an overall lower sensitivity field correlation and a third of the Miura-ori correlation’s variance (Fig. 5c). These characteristics of the MCP-optimized transducer translate to the high reconstruction accuracy on a synthetic target (Fig. 5d). The MCP-optimized pattern achieves a lower $\:{L}_{2}\text{}$error and a higher SSIM confirming its better image quality, compared to the Miura-ori based transducer.

Judging by these results, the MCP-based FOCUS design demonstrates remarkable generalization in image reconstruction across types of imaging targets, including point-scatterers, vessel-like images and 3D scatterers. It surpasses the benchmark provided by the validation objective in terms of SSIM, which struggles to accurately reconstruct images outside of the training set. The MCP-based FOCUS’s superiority can be attributed to two key factors. Firstly, the MCP does not necessitate a training set of imaging targets and optimizes the transducer geometry directly by introducing minimum coherence in the TIR, which translates to the objective of achieving an uncorrelated sensitivity field for best imaging performance. This optimization process results in an unbiased design that can reconstruct images effectively across target types. In contrast, the validation objective introduces a bias due to the reliance on the training set. Secondly, the MCP objective is computationally less expensive to evaluate, as it excludes the task of image reconstruction (see Methods). This optimization allows for more black-box evaluations in a shorter time frame, which ultimately contributes to the convergence of the optimization process.

### FOCUS robustness against noise

**Fig. 6:**
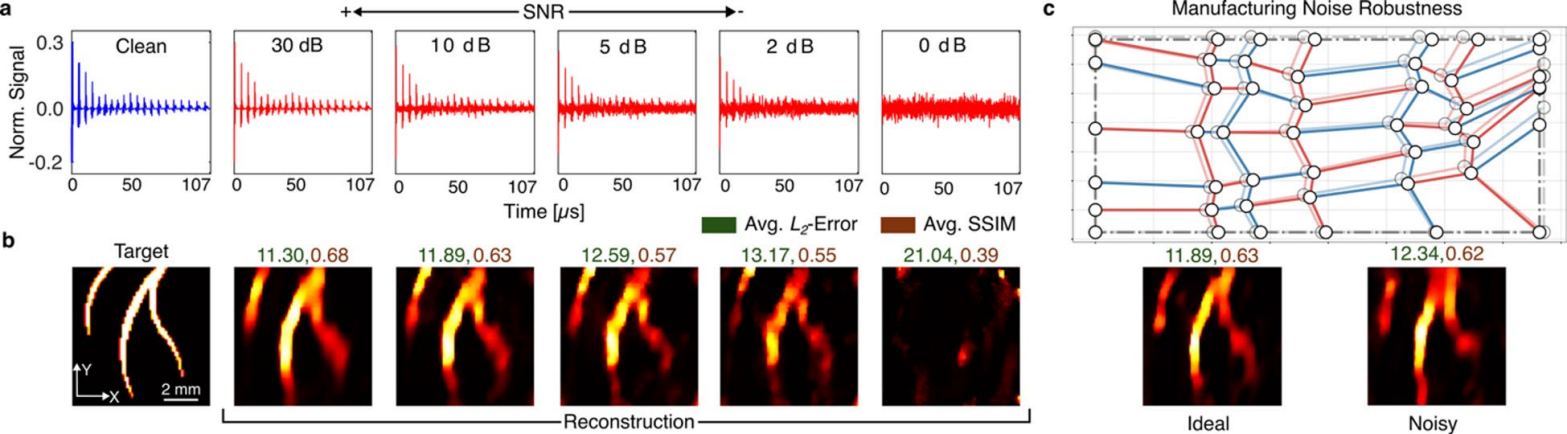
Robustness of FOCUS imaging to electrical and manufacturing noise. (**a**) Normalized signals $\:\overline{y}$ detected by the MCP-optimized FOCUS for different noise levels (see labels). (**b**) Reconstruction results for a vessel-like target under varying levels of noise. The leftmost image shows the ground truth imaging target, followed by reconstructions obtained from the corresponding noisy signals shown in (**a**). Average $\:L_2$-errors and average SSIM values over the test set are indicated by the colored labels. (**c**) MCP-optimized crease pattern with perturbation in terms of geometric imperfections overlaid on the original MCP-optimized pattern (with transparency). (**d**) Reconstructed vessel-like target using the ideal and perturbed FOCUS pattern in (**c**), both with 10 dB SNR electrical noise. On the test set (see Fig. 4a), the perturbed pattern yields an average $\:L_2$-error of 12.34 and an average SSIM of 0.62.

Electrical noise is unavoidable in practical ultrasound-mediated imaging. Thus, we tested FOCUS’ robustness against noise by simulating increasing magnitudes of electrical noise in term of signal-to-noise ratio (SNR) (Fig. 6a). FOCUS can still accurately reconstruct vessel-like targets even with an SNR of 2 dB.

To assess FOCUS’s tolerance to geometrical imperfections that may occur during manufacturing, we introduce Gaussian noise to the optimized sector angle variables $\:{\boldsymbol{\upalpha\:}}^{\mathrm{*}},\:{\mathbf{l}}^{*}$ (Fig. 6c) by sampling from $\:\mathcal{N}\left(\mathrm{0,3}\right)\:$and adding it to the first two sector angles in the lower-left corner of the L-shaped motif. By application of the panel compatibility theorem of the RFFQM (see Methods), the noise propagates though the complete crease pattern and perturbs the full design. The Field-II simulation is repeated and the image reconstruction performed for the perturbed crease pattern design. Despite these imperfections, the reconstructed image remains comparable to the noise-free pattern one. An evaluation of the imaging performance on the test set shows an increase in the $\:{L}_{2}$-error of only 0.45 and decrease in SSIM of 0.01 with the noisy transducer, implying strong robustness of the method to manufacturing imperfections.

## Discussion

We introduced a versatile simulation and novel inverse-design framework created to design advanced ultrasound/optoacoustic imaging devices. The FOCUS concept can achieve three-dimensional imaging with a single channel and a rigidly foldable origami mechanism. Choosing SDOF origami as the underlying mechanism of FOCUS enables a continuous change of the transducer’s surface geometry. This allows ultrasound sensing at multiple folding states that gathers enough information for reliable compressed-sensing imaging under the sparsity assumption of tissue. The non-trivial design choice of the origami was optimized to follow the introduced minimum coherence principle (Eq. (1)) of the transducer’s total impulse response. This principle is a necessary attribute of the single-channel transducer for accurate reconstruction of tissue using the proposed total variation least squares approach.

Our flexible framework can be adapted to different imaging scenarios, accommodating different targets, depths, and resolutions. FOCUS’ imaging capabilities can in principle be scaled by proportionally adjusting both the origami size and the central ultrasound wavelength (frequency), while the fine imaging resolution and the numerical stability of the reconstruction are influenced by the ultrasound bandwidth.

FOCUS exploits the transducer’s own surface instead of additional scatterers^12–15^ or reverberating cavities^16–19^, which consistently reduce the acoustic energy. Thus, our approach is expected to have a good pressure sensitivity. Despite the idealized conditions used in our simulations, FOCUS’ imaging performance is robust to electrical noise as well as geometric imperfections, which would be captured by the measurement of the imaging matrix using hydrophone calibration in a physical setup. The current ultrasound simulation does not incorporate inter-facet ultrasound reflections within the FOCUS transducer, which might appear at higher folding degrees. These reflections are expected to be captured by the calibration procedure and its impact in the image reconstruction should be clarified in the future.

Before a physical device can be built according to the FOCUS concept, a few manufacturing hurdles need to be tackled. First, it could be fabricated using piezo polymers attached to a rigidly foldable structural mesh that can be reliably and repeatably actuated. The actuation of the origami mechanism can be performed with small linear or rotary actuators along the corner vertex trajectories (Fig. S4, Supp. Movie S2) and must be synchronized with ultrasound generation/acquisition. Because of the mechanical actuation, imaging with a FOCUS device is expected to perform slower than array-based devices equipped with electronic steering, making it less suitable for monitoring rapid processes. Then, a calibration procedure should be performed to measure the imaging matrix for each folding state, which will capture the device’s uniqueness and use it to the image reconstruction’s advantage.

## Conclusion

In this computational work, we have demonstrated that single-channel compressive ultrasound sensing can be inversely designed using the rigid-folding principle of origami. By leveraging the complex surface kinematics across multiple folding states of the transducer, we showed in simulation its ability to recover high sensitivity in the pressure field despite using a single-data channel. This translates to an accurate and generalizable image reconstruction performance using the FOCUS concept across diverse imaging targets in both two and three dimensions while maintaining robustness against simulated electrical noise and geometrical imperfections. Although experimental realization will require advances in fabrication and actuation, the FOCUS concept offers a pathway toward compact and potentially wearable ultrasound/optoacoustic devices for chronic monitoring of diseases^36,37^. Beyond ultrasound, the presented work may also inspire new classes of origami-based sensing devices where the reconfigurable geometries can be leveraged for compressed acquisition of complex physical fields.

### Methods

#### FIELD-II ultrasound simulation

The ultrasound wave propagation is simulated using Field-II^38^, which computes the acoustic pressure field of a defined transducer geometry (see Supp. Information 1 for more details). The received acoustic pressure field of a specific FOCUS transducer folding state, parametrized by the design variables $\:\boldsymbol{\upalpha\:}\text{}$and $\:\mathbf{l}$ and the driving angle $\:\rho\:$, in response to a Dirac delta excitation is denoted by $\:\mathbf{h}\left(\boldsymbol{\upalpha\:},\:\mathbf{l},\rho\:\right)\in\:\text{}{\mathbb{R}}^{T\times\:{N}^{2}},\text{}$where $\:T\text{}$is the number of time samples and $\:{N}^{2}$ is the number of pixels in the FOV. Since an infinite bandwidth excitation pulse is unrealistic in a physical setup, $\:\mathbf{h}\left(\boldsymbol{\upalpha\:},\text{}\mathbf{l},\rho\:\right)$ is convolved with a finite bandwidth excitation pulse $\:{\mathbf{h}}_{\mathrm{e}}\in\:\:{\mathbb{R}}^{T}$. To account for the electromechanical coupling of the transducer’s piezo elements, another finite bandwidth convolution is applied in terms of the effective electrical impulse $\:{\mathbf{h}}_{\mathrm{e}\mathrm{r}}\in\:\:{\mathbb{R}}^{T}$. The excitation pulse and the electrical impulse response are modeled as Chebyshev-windowed cosine functions with a central frequency $\:{f}_{c}\mathrm{=}2\text{}\mathrm{M}\mathrm{H}\mathrm{z}$, which, when applied to the acoustic pressure field, define the transducer’s bandwidth $\:{f}_{b}\mathrm{=}1\text{}\mathrm{M}\mathrm{H}\mathrm{z}$ to mimic commercially available piezo transducers. The total impulse response (TIR) of a transducer in the $\:i$-th folding state $\:{\mathbf{A}}_{i}\left(\boldsymbol{\upalpha\:},\:\mathbf{l},\rho\:\right)\text{}$is calculated as

3 $$\:{\mathbf{A}}_{i}\left(\boldsymbol{\upalpha\:},\:\mathbf{l},\rho\:\right)=\mathbf{h}\left(\boldsymbol{\upalpha\:},\:\mathbf{l},\rho\:\right)*{\mathbf{h}}_{\mathrm{e}}*{\mathbf{h}}_{\mathrm{er}}$$.

The individual TIRs $\:{\mathbf{A}}_{i}\in\:\:{\mathbb{R}}^{T\times\:{N}^{2}}$of the $\:S$ folding states corresponding to the driving angles $\:\rho\:$ are then appended to form the stacked imaging matrix $$\:\stackrel{-}{\mathbf{A}}=\:{\left[\begin{array}{ccc}{\mathbf{A}}_{1}&\:\dots\:&\:{\mathbf{A}}_{S}\end{array}\right]}^{\mathrm{T}}\in\:{\mathbb{R}}^{ST\times\:{N}^{2}}$$.

By spatial convolution of the stacked TIRs with the acoustic source distribution in the tissue sample (imaging target $\:\mathbf{x}\in\:\:{\mathbb{R}}^{{N}^{2}}$) the measured voltage signal $$\:\stackrel{-}{\mathbf{y}}=\:{\left[\begin{array}{ccc}{\mathbf{y}}_{1}&\:\dots\:&\:{\mathbf{y}}_{S}\end{array}\right]}^{\mathrm{T}}\in\:{\mathbb{R}}^{ST}$$ is generated according to the linear model $\:\stackrel{-}{\mathbf{y}}=\stackrel{-}{\mathbf{A}}\mathbf{x}$.

We compute $\:\stackrel{-}{\mathbf{A}}$ for all two-dimensional imaging planes, with the field-of-view (FOV) in the lateral $\:xy$-plane over an area of 8 $\:\mathrm{m}\mathrm{m}$ × 8 $\:\mathrm{m}\mathrm{m}$ at a fixed imaging depth of 17 $\:\mathrm{m}\mathrm{m}$.

Since our approach relies on simulated data, we avoid using the same model for both the computation of the measurement vector $\:\stackrel{-}{\mathbf{y}}$ and the reconstruction $\:\widehat{\mathbf{x}}$ of the imaging target. This issue, known as the inverse crime^39^, is mitigated by using a different discretization of the FOV for the data generation model and reconstruction model. Specifically, $\:\stackrel{-}{\mathbf{A}}$ is computed on a FOV discretized with a pixel size of 80$\:{\upmu\:}\mathrm{m}$to simulate $\:\stackrel{-}{\mathbf{y}}$, while a separate version of $\:\stackrel{-}{\mathbf{A}}$, based on a coarser 100 $\:{\upmu\:}\mathrm{m}$ pixel grid, is used to reconstruct $\:\widehat{\mathbf{x}}$ during evaluation. To further prevent unrealistically optimistic results, Gaussian noise is added to the simulated measurement vector $\:\stackrel{-}{\mathbf{y}},$ resulting in a signal-to-noise ratio (SNR) of 10 dB. During the optimization using the MCP-based objective function in Eq. (1), a FOV of 20 $\:\mathrm{m}\mathrm{m}$ x 20 $\:\mathrm{m}\mathrm{m}$ with a pixel size of 200 $\:{\upmu\:}\mathrm{m}$ was used to enhance generalizability with a larger FOV.

In case of a three-dimensional FOV, the equivalent process is followed to acquire the acoustic pressure field and imaging matrix where the respective spatial dimensions are extended to the number of voxels in the FOV. In case of a cube-shaped FOV, this would result in $\:{N}^{3}$ voxels with a 3D imaging target $\:\mathbf{x}\in\:\:{\mathbb{R}}^{{N}^{3}}$, as depicted in Fig. 4d.

### Total variation least squares reconstruction

The image reconstruction is formulated as a regularized inverse problem, by imposing a sparsity bias on the estimation of $\:\mathbf{x}$. The aim is taken to minimize a joint objective combining the $\:{L}_{2}$-data fidelity term, $\:{L}_{1}$-sparsity, and total variation regularization. To achieve this, we employ a stochastic gradient descent optimizer with adaptive momentum^40^ (ADAM), since classical solvers for compressed sensing problems, such as basis pursuit^41^, show a slower convergence time. The optimization problem is defined as:

4 $$\:\widehat{\mathbf{x}}=\mathrm{arg}\underset{\mathbf{x}}{\mathrm{min}}{||\stackrel{\sim}{\mathbf{A}}\mathbf{x}-\stackrel{\sim}{\mathbf{y}}||}_{2}^{2}+{{\uplambda\:}}_{1}{||\mathbf{x}||}_{1}+{{\uplambda\:}}_{2}\mathrm{TV}\left(\mathbf{x}\right).$$

To ensure comparable optimization performance across different transducer geometries, the measurement matrix $\:\stackrel{-}{\mathbf{A}}$ is column wise normalized with respect to the $\:{L}_{2}$-norm, forming $\:\stackrel{\sim}{\mathbf{A}}$,such that $\:{||{\stackrel{\sim}{\mathbf{A}}}_{:,\:\:j}||}^{2}=\:1\:\forall\:\:{j}\:\in\:\:\left\{1,\:\dots\:,\:{N}^{2}\right\}.$ Similarly, the measurement vector $\:\stackrel{\sim}{\mathbf{y}}=\frac{\mathbf{y}}{{||\mathbf{y}||}_{2}}$ represents the unit $\:{L}_{2}$-norm transformation of $\:\mathbf{y}$. To avoid local minima, we randomly extract mini-batches of dimension 1000 from the data-vector $\:\stackrel{\sim}{\mathbf{y}}$. The step size (learning rate) for each iteration is set to $\:=2*{10}^{-5}$ and the stopping criteria are enforced when the minimization does not improve for 500 consecutive iterations. The assumed sparsity of $\:\mathbf{x}$ is enforced by applying $\:{L}_{1}$-regularization with $\:{\lambda\:}_{1}\mathrm{=}{10}^{\mathrm{-}4}$. To ensure local smoothness while preserving edges in the reconstruction, a total variation regularization $\:\mathrm{T}\mathrm{V}\left(\mathbf{x}\right)$ is employed with $\:{\lambda\:}_{2}\mathrm{=}{10}^{\mathrm{-}4}$ (see Supp. Information 2). Additionally, a rectified linear unit is applied to $\:\mathbf{x}$ to further promote sparsity and enforce $\:\:\mathbf{x}\in\:\:{\mathbb{R}}^{+}$ after each mini-batch iteration. Finally, to ensure interpretability the output of the optimization $\:\mathbf{x}$ was normalized to $\:\mathbf{x}\in\:\left[\mathrm{0,1}\right]$.

### RFFQM parametrization

We design the crease pattern based on a 5 × 5 grid of internal vertices (Fig. 2a), yielding a compact, 30-dimensional continuous design space for the RFFQM. This space comprises 18 independent sector angle variables $\:\boldsymbol{\upalpha\:}$, distributed over the nine internal degree-4 vertices of the L-shaped motif. The complementary 18 sector angles are then calculated using Kawasaki’s condition^42^, guaranteeing local flat foldability. The remaining 12 design variables correspond to the crease line lengths $\:\mathbf{l}$ within the L-shaped motif (highlighted by bold blue lines in Fig. 2a). Subsequently, the panel-compatibility theorem is employed to derive the complete remaining coupled geometry of the crease pattern. Certain configurations of sector angles $\:\boldsymbol{\upalpha\:}$ and crease lengths $\:\mathbf{l}$, however, may violate panel compatibility, resulting in intersecting crease lines. Such infeasible origami configurations are penalized during optimization by assigning a high objective function value, $\:\mathcal{L}\left(\boldsymbol{\upalpha\:},\:\mathbf{l}\right)={10}^{6}$ (see Supp. Information 4). Additionally, to enforce a rectangular boundary shape in the RFFQM (Fig. 2), the lengths of crease lines connected to boundary vertices are scaled such that their vertices align precisely into a rectangle. The scale factors for each boundary side depend directly on the boundary crease lengths within the L-shaped motif, resulting in one free scaling variable per side of the rectangle.

### Transducer alignment

The alignment of the origami-based transducer within a global reference frame is performed by first ensuring that the four corner vertices of the rectangular crease pattern remain approximately coplanar with the $\:xy$-plane of the global frame (see Supp. Information 3 for more details) and the rectangular boundary approximately collinear with the $\:x$- and $\:y$-axis. Maintaining coplanarity of the corner vertices throughout the folding motion greatly simplifies the tracking of those corner vertex trajectories, facilitating the design of actuation mechanisms based on these simple and predictable paths. A visualization of the corner vertex trajectories for the MCP-optimized origami is provided in the Supplementary Fig. S4. After alignment, the origami-based transducer is subsequently centered about the origin and scaled with a factor $\:s$ such that its projected area onto the $\:xy$-plane does not exceed $\:400{\mathrm{m}\mathrm{m}}^{2}$ for the folding state $\:\rho\:\mathrm{=}5^\circ\:$.

### Mesh adaptive directed search (MADS) optimizer

Both the MCP objective (Eq. (1)) and the validation objective (Eq. (2)) are optimized using the Mesh Adaptive Direct Search (MADS) algorithm^34^. A gradient-free optimizer is chosen due to the complexity and inherent non-differentiability involved in evaluating the black-box objective functions. The MADS optimization is initialized using a Latin-hypercube sampling performed on a mesh comprising $\:{10}^{5}$ evaluation points in the design space $\:\boldsymbol{\upalpha\:},\:\mathbf{l}$ in addition to the design choice corresponding to a standard Miura-ori pattern as visualized in Fig. 3d. The search directions within the pattern search are determined using the exploration in $\:2D$ orthogonal directions, with $\:D$ representing the number of design variables. To enhance global optimization capability and avoid local minima, an additional variable neighborhood search is used with an activation ratio of 0.8. Optimization is terminated upon satisfying either one of the following three criteria: A minimum mesh size reaching $\:{10}^{\mathrm{-}5}$, a maximum of $\:{10}^{6}$ function evaluations, or exceeding a computational runtime limit of 72 h. All optimizations were executed on a high-performance computing (HPC) cluster utilizing 10 CPU cores to enable parallel execution, particularly for the computationally intensive total variation least squares reconstruction in the validation objective. Given the inherently stochastic nature of the MADS algorithm, the optimization process was repeated across six independent runs, each initiated with distinct random seeds (see Supp. Table S1). The run yielding the best overall performance on the test set was selected and presented as the representative optimized origami structure within the main text.

### FOCUS optimization constraints

The FOCUS optimization incorporates four design constraints in addition to minimizing the objective functions $\:\mathcal{L}$ or $\:{\mathcal{L}}_{\mathrm{v}\mathrm{a}\mathrm{l}}$. These constraints ensure specific geometric and kinematic properties of the origami during folding, ensure ease of actuation and restrict the folding motion of the transducer design to a predefined space. The first constraint limits the dihedral angles between adjacent facets in the folded state of the origami to a maximum of 120°. Beyond this threshold, structural effects such as face thickness and imperfect crease compliance become increasingly significant, widening the simulation to reality gap.

The second constraint limits the crease lengths of the origami pattern to the interval $\:0.4s<l<2.0s$, where $\:s$ represents the scaling factor derived from the global frame alignment.

The third constraint limits the deformation of the origami structure along the $\:z$-axis to a maximum height of $\:7\mathrm{m}\mathrm{m}$ across all folding states, enabling the integration of the transducer within a compact, wearable form factor.

Finally, the fourth constraint ensures accurate global alignment of the corner vertices with the $\:xy$-plane by limiting the residual deviations of the corner vertices from the fitted reference plane. To facilitate practical implementation, this constraint is formulated such that the maximum vertex displacement of the corner vertices along the $\:z$-axis does not exceed $\:2\mathrm{m}\mathrm{m}$.

## Supplementary Information

Below is the link to the electronic supplementary material.

Supplementary Material 1

## Data Availability

Data and code are available from the corresponding authors upon reasonable request.
