# Supplementary material for: Computational design of foldable origami-based compressive ultrasound sensing
Source: Sci Rep. 2026 Jan 31;16:6839. doi: 10.1038/s41598-026-37215-5 (PMC12916773; doi:10.1038/s41598-026-37215-5)
Supplement: Supplementary file 1 — Supplementary Material 1 [file 41598_2026_37215_MOESM1_ESM.pdf]

Supplementary Information

Computational Design of Foldable Origami-based Compressive Ultrasound Sensing

Nicolas Hochuli, Tim Wünsch, Weiye Li, Xiaohan Han, Daniel Razansky, Tino Stanković*, Héctor Estrada*

*Corresponding authors. hector.estrada@pharma.uzh.ch and tinos@ethz.ch

The supplementary information includes:

Methods:

1. Ultrasound Simulation Description
2. Total Variation Regularization in 2D and 3D
3. Global Origami Alignment
4. Optimization-based Inverse Design Formulation
5. Acoustic Sensitivity Field of the MCP-optimized FOCUS Transducer

   Supplementary References

Figures, Tables, Movies and .fold files:

Table S1: L2 Errors and SSIM of all Runs
Fig. S1: Sensitivity Analysis of Different Folding Degree Combinations
Fig. S2: Training Set for Validation Objective
Fig. S3: Detailed Optimized Crease Patterns
Fig. S4: Folded Origami Configuration with Intended Actuation Paths
Movie S1: animation of US field of optimized origami
Movie S2: Origami folding motion with actuation paths
.fold files: MCP-optimized and validation-optimized crease pattern as .fold files.

1. Ultrasound Simulation Description

We provide a detailed description of the ultrasound model used in Field-II [1] which computes the acoustic pressure field and total impulse response (TIR) $\mathbf{A}$ of a FOCUS transducer with a predefined surface geometry. For sake of simplicity, the description is outlined for a two-dimensional imaging plane but follows the equivalent process in a three-dimensional imaging setting. The acoustic pressure field of a transducer is mathematically described as the linear map of an excitation Dirac pulse δ into a function of the scalar acoustic pressure $h(\mathbf{r}_1, t) \in \mathbb{R}$ at any point $\mathbf{r}_1 \in \mathbb{R}^3$ in the Field-of-View (FOV) over time t. The pressure is calculated as the integral of all spherical waves originating from all points on the transducer surface Q as:

$$h(\mathbf{r}_1, t) = \int_Q \frac{\delta\left(t - \frac{|\mathbf{r}_1 - \mathbf{r}_2|}{c}\right)}{2\pi|\mathbf{r}_1 - \mathbf{r}_2|} \mathrm{d}\mathbf{r}_2 \tag{1}$$

Where $|\mathbf{r}_1 - \mathbf{r}_2|$ is the distance from the transducer at position $\mathbf{r}_2 \in Q$ to the field point $\mathbf{r}_1$, $\delta(t)$ is the Dirac delta function and c is the speed of sound constant. The transducer surface Q in the FOCUS framework is defined as an RFFQM [2] origami that is parametrized using the set of design variables $\boldsymbol{\alpha}, \mathbf{l}$ and a specific driving angle ρ (see main text). This renders the acoustic pressure a function of the design variables and driving angle in terms of $h(\boldsymbol{\alpha}, \mathbf{l}, \rho, \mathbf{r}_1, t)$. The acoustic pressure is evaluated on a field by extending the vector $\mathbf{r}_1$ to a set of field locations spanning a FOV of $N \times N$ pixels in the xy-plane beneath the transducer in terms of $\left\{\mathbf{r}_1^{(j)}\right\}_{j=1}^{N^2} \subset N^2 \times \mathbb{R}^3$. The pressure evolution is evaluated over a finite time horizon $\mathbf{t} = \left\{0, \dots, \frac{T}{f_s}\right\}$ where the sampling frequency is $f_s = 20\ \mathrm{MHz}$ and the number of samples T is depending on the maximum time-of-flight distance between the FOV and transducer. Given the defined evaluation field, we can formulate a tensorized acoustic pressure field as:

$$\mathbf{h}(\boldsymbol{\alpha}, \mathbf{l}, \rho)=\begin{bmatrix} h\left(\mathbf{r}_1^{(1)}, t_1\right) & \cdots & h\left(\mathbf{r}_1^{(N^2)}, t_1\right) \\ \vdots & \ddots & \vdots \\ h\left(\mathbf{r}_1^{(1)}, t_T\right) & \cdots & h\left(\mathbf{r}_1^{(N^2)}, t_T\right) \end{bmatrix} \in \mathbb{R}^{T\times N^2} \tag{2}$$

The retrieved, idealistic, pressure field is further convolved with a realistic excitation pulse represented as a Chebyshev window [3] $w(t)$ with 3.2 periods and a central frequency f_c=2 MHz. Evaluating the window on the discretized time vector $\mathbf{t}_\mathrm{e}=\left\{0, \ldots, \frac{k_\mathrm{e}}{f_s}\right\}$ with a time horizon $k_\mathrm{e}=\frac{3.2}{f_c}$, yields an excitation pulse

$$\mathbf{h}_\mathrm{e}=\left[\cos\left(2\pi f_c t_{\mathrm{e},1}\right)\cdot w\left(t_{\mathrm{e},1}\right), \quad \ldots \quad, \cos\left(2\pi f_c t_{\mathrm{e},T_\mathrm{e}}\right)\cdot w\left(t_{\mathrm{e},T_\mathrm{e}}\right)\right]^\mathrm{T} \tag{3}$$

with a total of T_e samples. Analogously, we define the effective electrical impulse response, which accounts for electromechanical couplings in the transducer, over 4.7 periods. This leads to $\mathbf{t}_\mathrm{er}=\left\{0, \ldots, \frac{k_\mathrm{er}}{f_s}\right\}$ with $k_\mathrm{er}=\frac{4.7}{f_c}$ and a total of T_er samples, yielding an effective bandwidth of $1\ \mathrm{MH}z$:

$$\mathbf{h}_\mathrm{er}=\left[\cos\left(2\pi f_c t_{\mathrm{er},1}\right)\cdot w\left(t_{\mathrm{er},1}\right) \quad \ldots \quad \cos\left(2\pi f_c t_{\mathrm{er},T_\mathrm{er}}\right)\cdot w\left(t_{\mathrm{er},T_\mathrm{er}}\right)\right]^\mathrm{T} \tag{4}$$

Both pulses $\mathbf{h}_\mathrm{e}$ and $\mathbf{h}_\mathrm{er}$ and convolved with the acoustic pressure field $\mathbf{h}(\boldsymbol{\alpha}, \mathbf{l}, \rho)$ vector over the temporal dimension to form the total impulse response (TIR) which is used as the imaging matrix:

$$\mathbf{A}(\boldsymbol{\alpha}, \mathbf{l}, \rho)=\mathbf{h}(\boldsymbol{\alpha}, \mathbf{l}, \rho) * \mathbf{h}_\mathrm{e} * \mathbf{h}_\mathrm{er}. \tag{5}$$

In case of ultrasound simulations in three dimensions, we follow the same procedure as outlined for 2D pixels by extending the spatial dimension to the number of voxels N^3 for a cube-like FOV.

2. Total Variation Regularization in 2D and 3D

The following section discusses the definition of the isotropic total variation regularization $\mathrm{TV}(\mathbf{x})$ used in the total variation least squares reconstruction. Since the reconstruction algorithm uses gradient-based optimization, the total variation derivative is required.

This quantity has been presented for the 2-dimensional scenario in the work by Zhou et al.[4]. The non-trivial extension to volumetric 3D information (required in Fig. 4d) is presented here. The total variation over a volume $\mathbf{x} \in \mathbb{R}^{N\times N\times N}$ with index i, j, k in each dimension is defined as,

$$\mathrm{TV}(\mathbf{x})=\sum_{i,j,k}\sqrt{\left(x_{i+1,j,k}\text{-}x_{i,j,k}\right)^2+\left(x_{i,j+1,k}\text{-}x_{i,j,k}\right)^2+\left(x_{i,j,k+1}\text{-}x_{i,j,k}\right)^2+\varepsilon} \tag{6}$$

with $\varepsilon=10^{-9}$. Then, the spatial gradient can be formulated according to Rudin et al. [5]:

$$\nabla\mathrm{TV}(\mathbf{x})=\nabla\cdot\left(\frac{\nabla\mathbf{x}}{\sqrt{|\nabla\mathbf{x}|^2+\varepsilon}}\right) \tag{7}$$

The explicit gradient formulation used in the implementation for any individual pixel location indexed by the subscript i, j, k is then given by:

$$\begin{aligned}
&\nabla\mathrm{TV}(\mathbf{x})_{i,j,k}\\
&= \frac{\left(x_{i,j,k}-x_{i-1,j,k}\right)+\left(x_{i,j,k}-x_{i,j-1,k}\right)+\left(x_{i,j,k}-x_{i,j,k-1}\right)}{\sqrt{\left(x_{i,j,k}-x_{i-1,j,k}\right)^2+\left(x_{i,j,k}-x_{i,j-1,k}\right)^2+\left(x_{i,j,k}-x_{i,j,k-1}\right)^2+\varepsilon}}\\
&- \frac{x_{i+1,j,k}-x_{i,j,k}}{\sqrt{\left(x_{i+1,j,k}-x_{i,j,k}\right)^2+\left(x_{i+1,j,k}-x_{i+1,j-1,k}\right)^2+\left(x_{i+1,j,k}-x_{i+1,j,k-1}\right)^2+\varepsilon}}\\
&- \frac{x_{i,j+1,k}-x_{i,j,k}}{\sqrt{\left(x_{i-1,j+1,k}-x_{i-1,j,k}\right)^2+\left(x_{i,j+1,k}-x_{i,j,k}\right)^2+\left(x_{i,j+1,k}-x_{i,j+1,k-1}\right)^2+\varepsilon}}\\
&- \frac{x_{i,j,k+1}-x_{i,j,k}}{\sqrt{\left(x_{i-1,j,k+1}-x_{i-1,j,k}\right)^2+\left(x_{i,j-1,k+1}-x_{i,j-1,k}\right)^2+\left(x_{i,j,k+1}-x_{i,j,k}\right)^2+\varepsilon}}
\end{aligned} \tag{8}$$

To enable the computation of both forward and backward finite differences at all voxels, including boundaries, the volume is padded with boundary values equally for two voxels in each spatial

dimension. This ensures consistent access to neighboring values for gradient computation throughout the entire domain.

3. Global Origami Alignment

The alignment of the origami in the global frame according to Fig. 2c in the main text is outlined in this section. We begin the alignment by first fitting a plane through the four corner vertices of the origami in a least-squares sense. The resulting plane, defined by the equation $ax+by+cz=0$, provides the normalized normal vector $\mathbf{n}=[a,b,c]^T$ which is then aligned with the positive z-axis of the global reference frame. If the vector $\mathbf{n}$ initially points in the negative z-direction, its direction is inverted to ensure consistency. The rotation axis required to align the origami's normal vector $\mathbf{n}$ with the z-axis is computed as $\mathbf{q}=\frac{\mathbf{n}\times\mathbf{e}_z}{\|\mathbf{n}\times\mathbf{e}_z\|}$ where $\mathbf{e}_z$ is the unit vector along the z-axis and the rotation angle around $\mathbf{q}$ is computed as $\theta_1=\arccos\left(\mathbf{n}\cdot\mathbf{e}_z\right)$. The corresponding rotation matrix $\mathbf{R}$ is then obtained using Rodrigues' rotation formula. We first construct the skew-symmetric matrix:

$$\mathbf{K}=\begin{bmatrix} 0 & -q_z & q_y \\ q_z & 0 & -q_x \\ -q_y & q_x & 0 \end{bmatrix} \quad (9)$$

and then compute $\mathbf{R}=\mathbf{I}+(\sin\theta_1)\mathbf{K}+(1-\cos\theta_1)\mathbf{K}^2$ where $q_{x,y,z}$ define the respective components of $\mathbf{q}$ and $\mathbf{I}$ the identity matrix. The rotation matrix $\mathbf{R}$ is applied to all 3D vertex positions $\mathbf{v}_j$ in $\mathcal{V}=\{\mathbf{v}_j \mid j=1,\dots,V\}$, where V defines the number of vertices in the origami. The alignment of the rectangular boundary of the origami with the x-axis and y-axis (see Fig. 2c in main text) is achieved by further rotating the origami around the z-axis of the global frame such that the connection between the lower-left and lower-right corner vertex (see corners in Fig. 2a in main text) is collinear with the x-axis in the projection to the xy-plane. Mathematically, if $\mathbf{v}_{c,1}$ and $\mathbf{v}_{c,2}$ are the lower-left and lower-right corner vertices in the crease pattern, then the angle of rotation is $\theta_2=\operatorname{arctan2}\left(\frac{\mathbf{v}_{c,2,y}-\mathbf{v}_{c,1,y}}{\mathbf{v}_{c,2,x}-\mathbf{v}_{c,1,x}}\right)$ where the extended subscript implies the x or y component of the vector. The negative angle is then used in the standard rotation matrix formulation about the z-axis $\mathbf{R}_z(-\theta_2)$ which is applied to all positions $\mathbf{v}_j$ in $\mathcal{V}=\{\mathbf{v}_j \mid j=1,\dots,V\}$. Subsequently, the

origami is centered in the global frame by computing the centroid μ_c of the four corner vertices and subtracting it from all $\mathbf{v}_j$ in $\mathcal{V}$. As a last step, to ensure that the projected area of the origami onto the global xy-plane does not exceed $400\ \mathrm{mm}^2$ for the folding state ρ=5°, we extract the maximum vertex distance p_x, p_y in both x and y direction in $\mathcal{V}$, respectively. The vertex positions are then scaled accordingly with a factor $s=\frac{20\ \mathrm{mm}}{\max(p_x,p_y)}$.

4. Optimization-based Inverse Design Formulation

The FOCUS transducer design is formally set up as a constrained continuous non-linear program in a parametrized 30-dimensional design space. This space comprises 18 independent sector angle variables $\boldsymbol{\alpha}$, distributed over the nine internal degree-4 vertices of the L-shaped motif in the RFFQM, where sector angles are bounded in the range $20° < \alpha_i < 160°$. The remaining 12 design variables correspond to the crease line lengths $\mathbf{l}$ within the L-shaped motif. The presumably non-convex and non-linear objective function to be minimized is denoted as $\mathcal{L}(\boldsymbol{\alpha}, \mathbf{l})$. We additionally define a set of five explicit non-linear geometric constraints $g_i(\boldsymbol{\alpha}, \mathbf{l}) \leq 0$. The optimization problem is then stated as:

$$\min_{\boldsymbol{\alpha},\mathbf{l}} \mathcal{L}(\boldsymbol{\alpha}, \mathbf{l}) \tag{10}$$

$$\text{s.t} \qquad g_1: \frac{1}{|P|}\sum_{\rho\in P}\frac{|\rho| - \bar{\rho}}{\bar{\rho}} \leq 0$$

$$g_2: \frac{1}{|L|}\sum_{l\in L}\frac{l - \bar{l}_{\min}}{\bar{l}_{\min}} + \frac{\bar{l}_{\max} - l}{\bar{l}_{\max}} \leq 0$$

$$g_3: \frac{1}{|Z_{\mathrm{PTP}}|}\sum_{z\in Z_{\mathrm{PTP}}}\frac{z - \bar{z}}{\bar{z}} \leq 0$$

$$g_4: \frac{1}{|Z_{\mathrm{C,PTP}}|}\sum_{z\in Z_{\mathrm{C,PTP}}}\frac{z - \bar{z}_c}{\bar{z}} \leq 0$$

Here, g_1 corresponds to the constraint of maximum allowed dihedral angle ($\bar{\rho}$=$120°$) on any crease line in the origami during the complete folding motion. The set P contains the collection of all dihedral angles across all folding states. Constraint g_2 both relates to the minimum ($\bar{l}_{\min}$=$0.4s$) and maximum ($\bar{l}_{\max}$=$2.0s$) allowed length of the crease lines, hence L holds the set of crease line lengths where s is the scaling factor from the global alignment. Constraint g_3 corresponds to the maximum allowed deformation $\bar{z}$=7 mm of the origami along the z-axis,

where Z_{PTP} gathers the maximum peak-to-peak distance in the origami along the z-axis, for all folding states. Finally, g_4 accounts for the maximum residual of the corner vertices ($\overline{z}_c$=2 mm) from the coplanar state in the xy-plane with $Z_{\mathrm{C,PTP}}$ holding the maximum peak-to-peak distance of the origami corner vertices along the z-axis for each folding state.

Instead of introducing a large number of individual constraints for each element in the sets $P, L,$ $Z_{\mathrm{PTP}}, Z_{\mathrm{C,PTP}}$, the constraints are summed into single aggregate expressions g_i. To retain all information without losing sensitivity to individual constraint violations, a conditional assembly procedure is used for the sets $P, L, Z_{\mathrm{PTP}}, Z_{\mathrm{C,PTP}}$.

- For each set, we evaluate the corresponding individual constraint term of all elements.
- If any individual constraint term is positive (i.e., violates the constraint), we retain only the positive terms in the whole summation and discard all negative terms (i.e. those satisfying the constraint).
- If all terms are non-positive, we retain all terms in the summation.
- Each constraint is normalized using the number of terms in the summation (or set).

Thus, although each constraint is a single summation, it remains sensitive to any individual violation inside the respective set.

5. Acoustic Sensitivity Field of the MCP-optimized FOCUS Transducer

Supp. Movie 1. visualizes the acoustic sensitivity field of the MCP-optimized transducer over all folding states. The sensitivity field shows the normalized temporal TIR evolution in the range [0,1] at all points in the FOV located at a depth of 17 mm beneath the transducer. The visualization evolves in the time interval $t \in \left[0, \frac{T_i}{f_s}\right]$ for each folding state i of the origami in the set of driving angles $\rho \in \{2°, 4°, \dots, 40°\}$. This results in a high-fidelity simulation for a total of 20 folding states where each TIR evolution is appended after instantaneous folding. This leads to a total simulation time of $\sum_{i=1}^{S} \frac{T_i}{f_s} = 107.7 \mu\text{s}$.

Supplementary References

[1] J. A. Jensen, “Simulation of advanced ultrasound systems using Field II,” in *2004 2nd IEEE International Symposium on Biomedical Imaging: Nano to Macro (IEEE Cat No. 04EX821)*, Apr. 2004, pp. 636-639 Vol. 1. doi: 10.1109/ISBI.2004.1398618.

[2] F. Feng, X. Dang, R. D. James, and P. Plucinsky, “The designs and deformations of rigidly and flat-foldable quadrilateral mesh origami,” *J. Mech. Phys. Solids*, vol. 142, p. 104018, Sep. 2020, doi: 10.1016/j.jmps.2020.104018.

[3] F. J. Harris, “On the use of windows for harmonic analysis with the discrete Fourier transform,” *Proc. IEEE*, vol. 66, no. 1, pp. 51–83, Jan. 1978, doi: 10.1109/PROC.1978.10837.

[4] Y. Zhang, Y. Wang, and C. Zhang, “Total variation based gradient descent algorithm for sparse-view photoacoustic image reconstruction,” *Ultrasonics*, vol. 52, no. 8, pp. 1046–1055, Dec. 2012, doi: 10.1016/j.ultras.2012.08.012.

[5] L. I. Rudin, S. Osher, and E. Fatemi, “Nonlinear total variation based noise removal algorithms,” *Phys. Nonlinear Phenom.*, vol. 60, no. 1, pp. 259–268, Nov. 1992, doi: 10.1016/0167-2789(92)90242-F.

Runs	MCP-based		Dataset-based	
	L_2-Error	SSIM	L_2-Error	SSIM
Seed 1	12.35	0.596	11.939	0.597
Seed 2	12.92	0.626	13.617	0.545
Seed 3	12.42	0.601	13.04	0.556
Seed 4	**11.89**	**0.629**	12.612	0.566
Seed 5	12.72	0.599	**11.697**	**0.597**
Seed 6	12.74	0.559	12.981	0.571
**Average**	**12.51**	**0.602**	12.647	0.572
**Standard Deviation**	0.37	0.025	0.723	0.021

Table S1. L2 Errors and SSIM of all Runs. Summary of all resulting metrics for the optimizations using different initial seeds. The metrics are reported in terms of the reconstruction performance of the MCP-based and validation-based optimized results using the L_2-Error and the SSIM averaged over the test set (see main text Fig. 4a and Table 1).

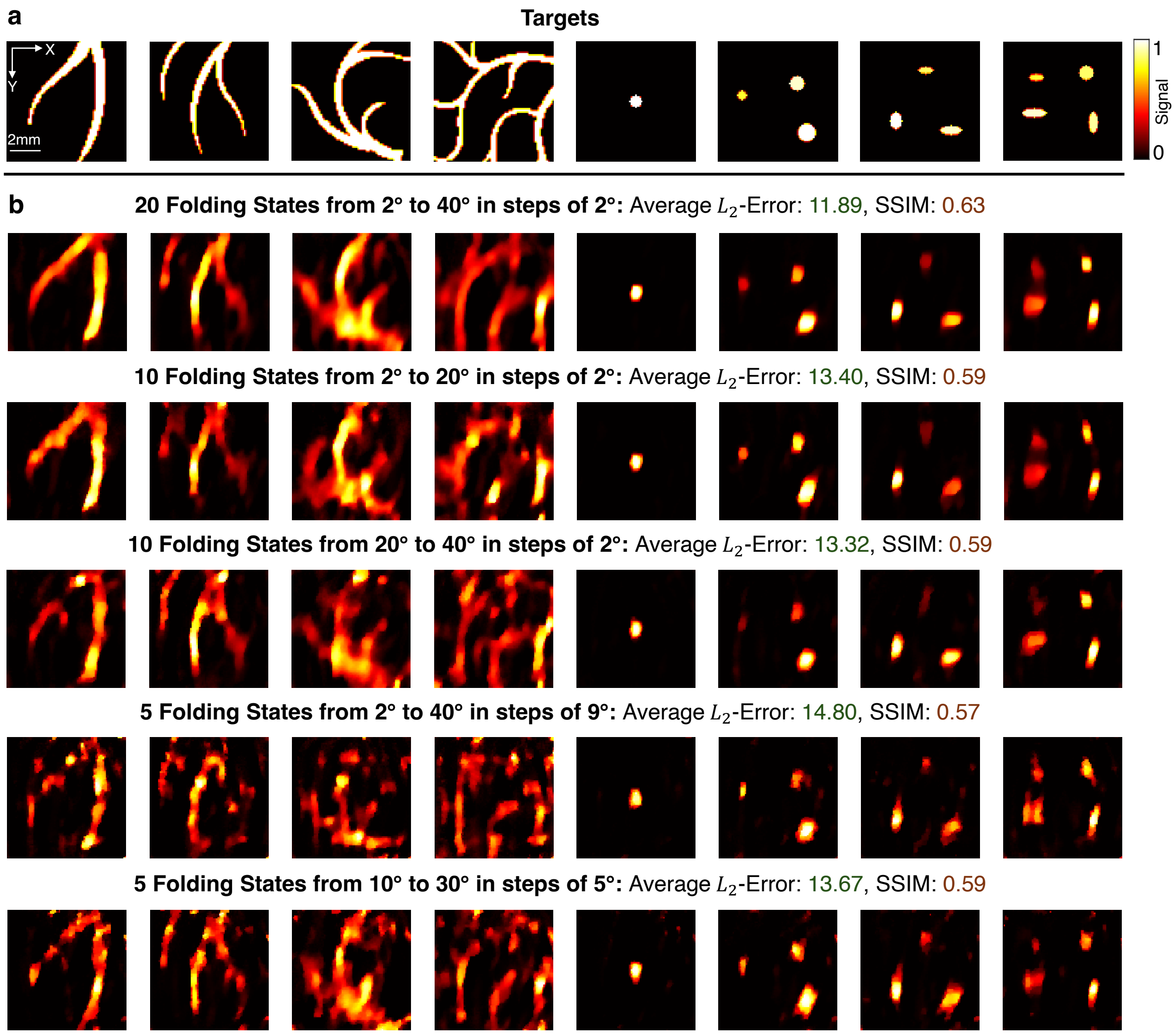

Fig. S1. Sensitivity Analysis of Different Folding Degree Combinations. The selection of driving angles varies the folded state of the SDOF origami and hence influences the TIR for the image reconstruction task on a test set. (**a**) The imaging targets in the test's set to be reconstructed using FOCUS. (**b**) The reconstruction with varying combinations of folding states of the MCP optimized transducer. The number of folding states used and which driving angle ρ that is used at each state is described in the header of each row. The corresponding metrics are reported as an average over the test set. It is visible that fewer folding states decrease the performance, while the choice of the range of the driving angles has less impact than the number of folding states.

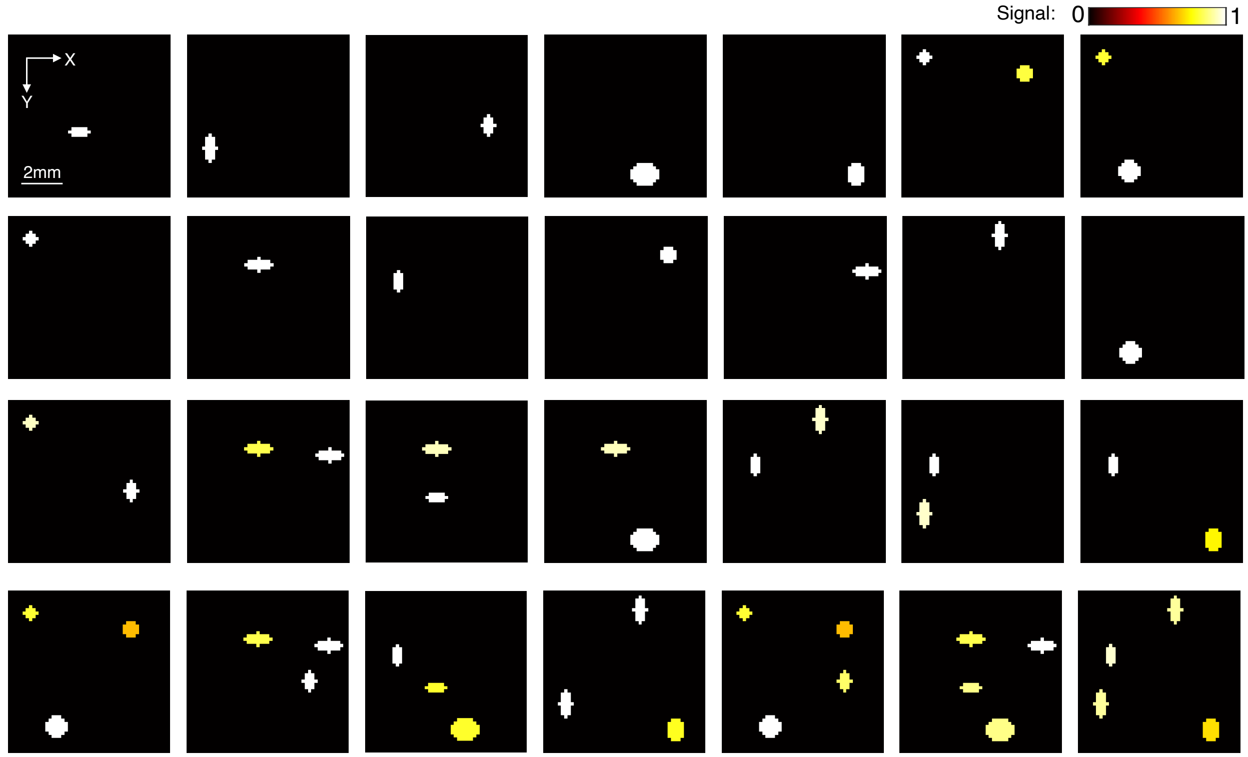

Fig. S2. Training Set for Validation Objective. The training set with 28 imaging targets consisting of randomly aligned scatterers in the FOV. The number of scatterers varies from one to four. The scatterers have random ellipsoid shape and size, and the color implies the relative acoustic attenuation coefficient of the scatterer. This training set is used in the optimization using the validation objective $\mathcal{L}_{\mathrm{val}}$.

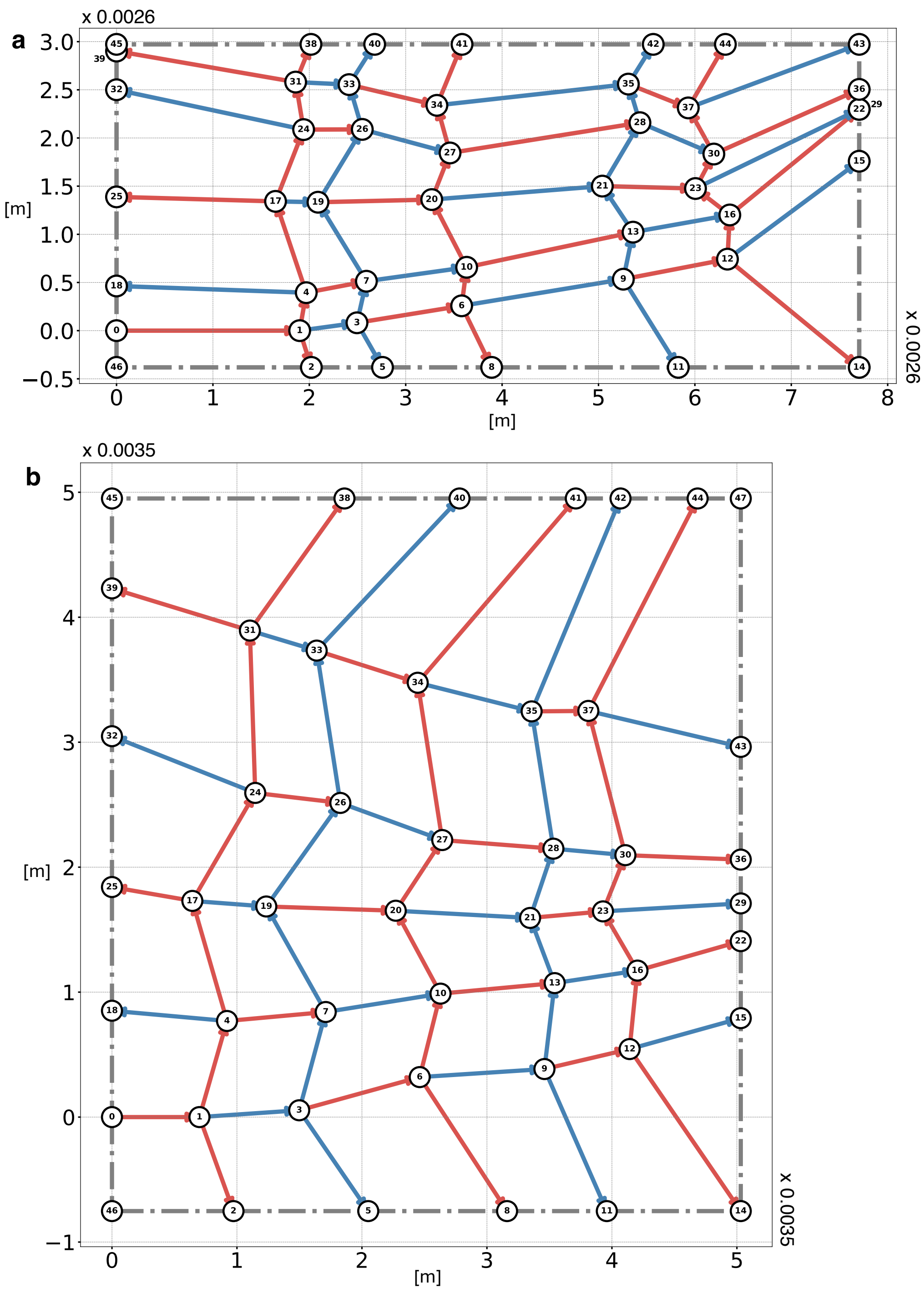

Fig. S3. Detailed Optimized Crease Patterns. Both crease patterns optimized using the MCP objective and the data validation objective. The scale of the crease pattern and vertex indices are annotated in the figures. The colors of the crease lines represent mountain/valley assignment. Red represents mountain fold and blue valley fold. Both crease patterns can be found in the supplementary material as .fold files. (**a**) The MCP optimized crease pattern. (**b**) The crease pattern optimized using the validation objective.

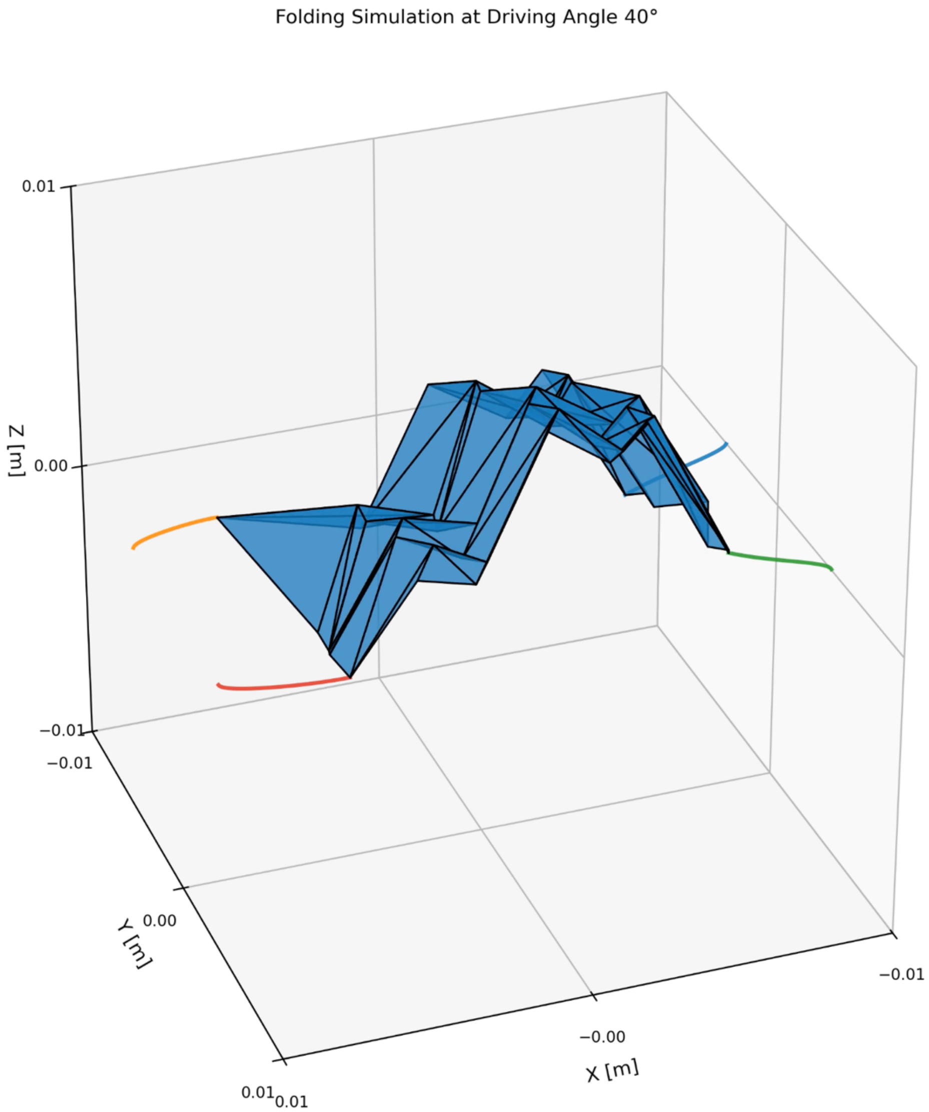

Fig. S4: Folded Origami Configuration with Intended Actuation Paths. Detailed triangulated FOCUS configuration of the MCP-optimized origami (Fig. 3a) at driving angle $\boldsymbol{\rho}$=$\mathbf{40}$°. The intended actuation paths of the corner vertices are marked with bold lines. A potential actuation mechanism should follow the paths using tracks with linear or rotary actuators, to fold the origami. The origami folds from $\boldsymbol{\rho}$=$\mathbf{5}$° to $\boldsymbol{\rho}$=$\mathbf{40}$° along these paths with its corner vertices. A movie with the full folding motion is available as Supp. Movie 2.
